# The Effectiveness of Pattern Scanning Laser Trabeculoplasty as an Additional Treatment for the Patients of Open-Angle Glaucoma Receiving Full Ocular Hypotensive Medications

**DOI:** 10.3390/jcm13113266

**Published:** 2024-05-31

**Authors:** Yosuke Ueno, Yusuke Haruna, Mami Tomita, Atsushi Sakai, Shogo Ogawa, Shigeru Honda

**Affiliations:** Department of Ophthalmology and Visual Sciences, Graduate School of Medicine, Osaka Metropolitan University, Osaka 545-8585, Japan; m21679m@omu.ac.jp (Y.U.);

**Keywords:** glaucoma, pattern scanning laser trabeculoplasty, full ocular hypotensive medications

## Abstract

**Objectives**: Our purpose was to examine the effectiveness of pattern scanning laser trabeculoplasty (PSLT) as an additional treatment for patients of open-angle glaucoma (OAG) receiving maximized ocular hypotensive medications (OHM). **Methods**: A total of 40 eyes of 33 patients (average age 72.7 ± 10.7 years) who had not previously undergone open glaucoma surgery or laser trabeculoplasty and were treated with maximized OHM between June 2018 and March 2022 were included. A 360-degree PSLT was conducted, and postoperative intraocular pressure (IOP) and survival curves at 1, 3, 6, 9, and 12 months were evaluated. **Results**: According to the Kaplan–Meier survival analysis, the average survival time was 8.1 months and the survival rate at 12 months was 0.55, with death defined as postoperative IOP reduction of less than 10% or requiring additional treatment. The average survival time was 4.9 months and the survival rate at 12 months was 0.28, with death defined as postoperative IOP reduction of less than 20% or requiring additional treatment. Nine eyes showed increased IOP (three eyes) or worsened visual field (six eyes) during the course and underwent additional open glaucoma surgery. In the 31 eyes which received no additional treatment after PSLT, the mean preoperative IOP was 18.5 ± 3.9 mmHg, which reduced to 15.3 ± 4.1 mmHg (*p* = 1.62 × 10^−6^), 15.5 ± 3.4 mmHg (*p* = 1.51 × 10^−5^), 15.7 ± 4.0 mmHg (*p* = 1.75 × 10^−5^), 14.7 ± 4.38 (*p* = 2.89 × 10^−6^), and 15.0 ± 4.0 mmHg (*p* = 5.74 × 10^−9^) at 1, 3, 6, 9, and 12 months after PSLT, respectively. The IOP reduction rate one year after PSLT was 18.7%. Of the 31 eyes, 13 (42%) achieved a 20% reduction in IOP compared to the baseline. **Conclusions**: Adjunctive treatment with PSLT in OAG patients receiving maximized OHM may be effective over 12 months of follow-up.

## 1. Introduction

Recently, various modalities have been developed for treating glaucoma [1], but glaucoma is still one of the most common causes of premature blindness. The only established treatment for glaucoma is to lower intraocular pressure (IOP). The initial treatments for open-angle glaucoma (OAG) are usually an application of ocular hypotensive medications (OHMs), and if the IOP fails to be controlled by several combinations of OHM, then surgical treatments are considered. Laser trabeculoplasty (LTP) is one of the surgical treatments for OAG that is often performed in clinical practice [2,3]. To date, there have been several modalities developed as LTP. Wise et al. introduced argon laser trabeculoplasty (ALT) in 1979 [4], and Latina et al. proposed selective laser trabeculoplasty (SLT) in 1995 [5]. Pattern scanning laser trabeculoplasty (PSLT) using the PASCAL^®^ laser developed by TOPCON was proposed in 2006 [6]. PSLT is a treatment method that allows multiple coagulations to be made at once, and the irradiation area can be controlled by computer-based monitoring, which is likely easier than performing SLT. A pilot study reported an average IOP reduction rate of 24% 6 months after 532 nm wavelength PSLT [6]. Wong et al. reported that the IOP reduction rate 1 year after PSLT was 11.6% [7], and Mansouri et al. reported that the IOP reduction rate 1 year after PSLT was estimated to be 14% [8]. Although SLT using a Q-switched laser is likely the most commonly performed LTP in current clinical practice [9], several reports have described that the IOP lowering rate of 577 nm wavelength PSLT and SLT was equivalent after 6 and 12 months [10], which encouraged the use of PSLT than SLT as adjunctive therapy for lowering IOP because of its easiness. However, most previous reports administrated LTP only after the OHM had been washed out, or at a stage where the number of OHMs was not maximized. Although it is common to perform open glaucoma surgery for patients using maximized OHM to decrease IOP and maintain the visual field [1], there might be some situations where surgery cannot be performed right away (e.g., financial and scheduling issues for the patient; systemic condition of the patients; hospital circumstances; and especially during the coronavirus pandemic, when there were cases where non-life emergent surgery is not possible at all). In such situations, LTP could be considered an adjunctive therapy in ordinary clinical practices. Thus, the effects of PSLT in this condition are likely worth evaluating.

Here, we retrospectively investigate the effectiveness of 577 nm wavelength PSLT as an additional treatment for OAG patients receiving maximized OHMs.

## 2. Subjects and Methods

This study was approved by the Institutional Review Board at the Osaka Metropolitan University Graduate School of Medicine (No. 2023-116) and was conducted following the Declaration of Helsinki. All cases in this study were Japanese individuals recruited from the Department of Ophthalmology at Osaka Metropolitan University Hospital in Japan. Written informed consent for the use of ordinary clinical data in the following retrospective studies was obtained from all subjects on their first visit to the hospital, and an opt-out for this study was indicated on the department website after approval of the study by the Institutional Review Board at the Osaka Metropolitan University Graduate School of Medicine.

The records of consecutive OAG patients who had not previously undergone any glaucoma surgery or LTP and who received maximized OHMs for glaucoma between June 2018 and March 2022 were reviewed in this study. The maximized OHMs consist of combinations of 3 or more eye drops, including prostaglandin F_2α_ analogs, beta-blockers, carbonic anhydrase inhibitors, α_2_ adrenergic agonists, and Rho kinase inhibitors. The cases of OAG secondary to uveitis, steroid use, or neovascular glaucoma were excluded. Gonioscopically open angles were a requirement for study inclusion. All IOP measurements were performed using Goldmann applanation tonometry. Visual fields were assessed using a Humphrey field analyzer (HFA) in 21 eyes and Goldmann perimetry in 19 eyes every 3–6 months, or more frequently if a progression of visual field defects was suspected. The average MD in 21 eyes examined with HFA was −16.8 ± 8.2 dB before PSLT. Although the visual field measured with Goldmann perimetry could not be evaluated using MD values, they all exhibited visual field impairment with the V-4 isopter, which corresponds to a III or higher grade according to the Kosaki classification and, hence, did not fall under the early stage of glaucoma.

PSLT was performed as an additional treatment without reducing the number of OHMs used before the treatment. For PSLT, PASCAL Streamline 577^®^ (wavelength 577 nm) (TOPCON, Tokyo, Japan) was used. The treatment procedure was carried out according to the company’s instructions. A single mirror gonio laser lens (1× Indexing Lens; Ocular Instruments, Bellevue, WA, USA) was used to project and align the laser patterns onto the trabecular meshwork (TM). Laser power was titrated by placing a single laser spot (100 μm diameter) into the inferior quadrant at a 10 ms exposure duration. In all cases, a starting power level of 200 mW was chosen, and power was reduced or increased until a barely visible lesion (light blanching of TM) was achieved. In a majority of eyes, some degree of pigmentation was visible in the inferior chamber angle (where titration was performed). If there was no pigmentation, the PSLT procedure was performed with 400 mW. After titration, the power was maintained, but the pulse duration was automatically reduced to 5 ms to produce subvisible lesions. The 360° treatment of TM was administered in 32 steps, where each pattern was composed of 39 spots spanning 11.25° of the trabecular meshwork—three rows of 13 spots each (1152 in total), with zero spacing between the adjacent spots—and the contact lens was rotated every 11.25° after a segment was treated to maintain no overlap and no gaps in the treatment spots. Apraclonidine eye drops were administered 30 min before and after laser treatment to prevent a transient increase in IOP. Postoperatively, 0.1% fluorometholone eye drops were administered as an anti-inflammatory agent, and cases suspected of being steroid responders were given 0.1% bromfenac sodium hydrate eye drops for one week. PSLT was performed once and not repeated over 12 months of follow-up. The IOP was measured monthly, and if a high IOP (>21 mmHg) was found, it was measured again within a few weeks. If a continuous increase in IOP and/or a worsened visual field was found, additional open glaucoma surgery (trabeculotomy or trabeculectomy) was performed.

The primary outcome measure was the IOP reduction rate at 12 months postoperatively. Changes in the IOP values and survival curve at 1, 3, 6, 9, and 12 months postoperatively were also evaluated as the secondary outcomes. To investigate the changes in the IOP after PSLT, cases who underwent additional open glaucoma surgery during the follow-up period were excluded from the assessment.

For statistics, IBM, SPSS ver.24.0 software was used. Changes in the IOP were evaluated using a paired *t*-test. Survival analysis was assessed using the Kaplan–Meier survival analysis table. A *p*-value of less than 0.05 was considered to be statistically significant.

## 3. Results

The baseline characteristics of the participants are shown in Table 1. A total of 40 eyes of 33 patients were included in this study. The average age was 72.7 ± 10.7 years (57–89 years), 19 patients were male, and 14 patients were female. The disease types were as follows: 35 eyes had primary open-angle glaucoma (POAG) and 5 eyes had pseudoexfoliation glaucoma (PEG). The average preoperative IOP was 20.1 ± 4.9 mmHg, and the eye drop score (counting 1 for single agents, 2 for combination agents) was 4.1 ± 1.1. The PSLT irradiation conditions were as follows: the average number of coagulations was 1297, the average laser power was 338 mW, and the average irradiation energy was 1.69 mJ. As for postoperative complications, one patient (2.5%) showed a transient increase in IOP exceeding 5 mmHg compared to the preoperative level, and no other changes such as anterior iris adhesion were observed in any patient.

At 12 months after PSLT, 23 out of 40 eyes (57.5%) showed a reduction in IOP of 10% or more compared to the baseline, and 13 eyes (32.5%) showed a decrease of 20% or more. Of 40 eyes, 31 (77.5%) were followed up for one year without any changes in the eye drops or additional treatments such as open glaucoma surgery. The other nine eyes had increased IOP (three eyes) or a worsened visual field (six eyes) during the course and underwent additional open glaucoma surgery (trabeculotomy for 1 eye, Express^®^ device insertion for 1 eye, and trabeculectomy for 7 eyes); hence, they were excluded from the subsequent analyses.

**Figure 1:**
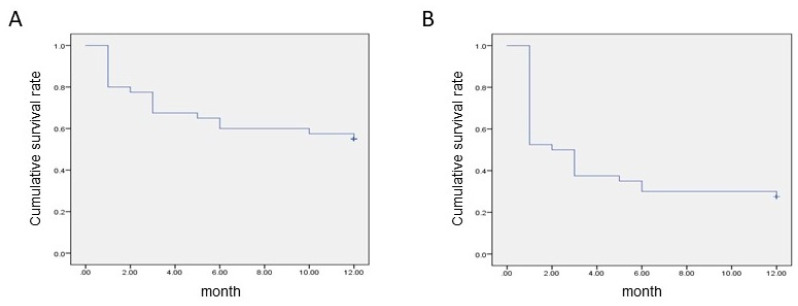
Results of Kaplan–Meier survival analysis if death was defined as the point in time when the rate of decrease in intraocular pressure was less than 10% (**A**) or 20% (**B**) twice in a row.

The results of Kaplan–Meier survival analysis of 40 eyes are shown in Table 2 and Figure 1. If death was defined as the point in time when the rate of decrease in IOP was less than 10% twice in a row, the survival rate gradually decreased over time, which resulted in an average survival time of 8.1 months, and the survival rate at 12 months was 0.55 (Table 2 and Figure 1A). If death was defined as the point in time when the rate of decrease in IOP was less than 20% on two consecutive occasions, the survival rate showed an acute decline to 0.52 a month after PSLT. Further, it decreased gradually until 12 months of follow-up was reached. Consequently, the average survival time was 4.9 months, and the survival rate at 12 months was 0.28 (Table 2 and Figure 1B).

To compare the baseline parameters between the drop-out and non-drop-out groups, there was no difference in age or sex. However, the mean baseline IOPs in the drop-out and non-drop-out groups were 25.8 ± 3.5 and 18.5 ± 3.9 mmHg, respectively, which are significantly different (*p* = 1.26 × 10^−5^). Moreover, the mean eye drop scores before PSLT were 4.8 ± 0.4 and 4.0 ± 1.2 in the drop-out and non-drop-out groups, respectively, which are also significantly different (*p* = 0.040).

**Figure 2:**
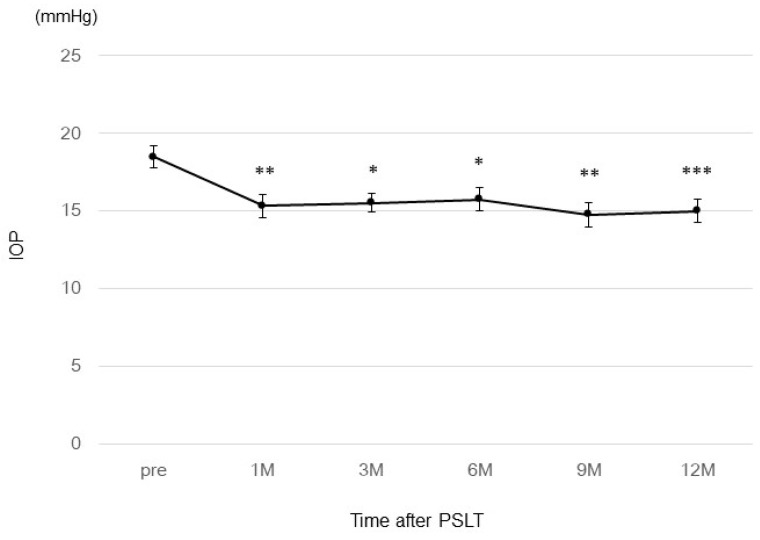
The chronological change in IOP in the 31 eyes which did not require any changes in eye drops or additional treatment after PSLT. * *p* < 1 × 10^−4^, ** *p* < 1 × 10^−5^, *** *p* < 1 × 10^−8^.

Figure 2 shows the chronological change in the IOP in the 31 eyes which did not require any change in eye drops or additional open glaucoma surgery after PSLT. In this group, the average IOP before PSLT was 18.5 ± 3.9 mmHg, and the average IOP and IOP reduction rate after PSLT were 15.3 ± 4.1 mmHg (*p* = 1.62 × 10^−6^), 17.3%; 15.5 ± 3.4 mmHg (*p* = 1.51 × 10^−5^), 16.2%; 15.7 ± 4.0 mmHg (*p* = 1.75 × 10^−5^), 15.1%; 14.7 ± 4.38 (*p* = 2.89 × 10^−6^), 20.5%; and 15.0 ± 4.0 mmHg (*p* = 5.74 × 10^−9^), 18.9% at 1, 3, 6, 9, and 12 months, respectively.

## 4. Discussions

In the present study, we have demonstrated that PSLT for the OAG cases receiving maximized OHM may have some treatment effects over 12 months. Namely, 58% of the eyes showed a 10% or greater reduction in IOP after PSLT, and 78% of the eyes avoided additional treatments after PSLT during this period.

A previous report mentioned that the IOP-lowering effect of PSLT was 24% after 6 months [6]. Another report showed that the IOP reduction rate 6 months after PSLT was approximately 19% [8]. They stated that the IOP reduction rate may have been modest due to the lower baseline IOP than that previously reported. In this study, the IOP reduction rate after PSLT was approximately 15% at 6 months and approximately 19% at 1 year in the eyes which required no additional treatments. The effect of PSLT on lowering IOP might be limited in the present study compared to the previous reports since we performed PSLT as an additional treatment for OAG patients who were being treated with maximized OHMs, who likely had more severe baseline conditions of glaucoma than those in the previous studies using PSLT. A previous report demonstrated that PSLT for uncontrolled ocular hypertension or POAG reduced the IOP from 20.3 mmHg to 15.9 mmHg (20.8% reduction) [11], which is consistent with our results. Kontić et al. reported that SLT reduced the mean IOP from 20.5 mmHg to 16.0 mmHg (21.9% reduction) at 12 months postoperatively in OAG patients receiving maximal medical therapy [12], which was almost equivalent to the result of this study. In contrast, 9 eyes out of 40 in the present study required additional glaucoma surgeries due to an elevation of IOP and/or a deterioration of visual field defects during the follow-up period, which likely affected the assessment of the mean IOP. Therefore, such cases were excluded from the analysis for the change in IOP after PSLT, and only 31 eyes could be assessed for the IOP reduction rate over 12 months. This might have caused a selection bias in calculating the IOP reduction rate after PSLT in this study. In the Kaplan–Meier survival analysis, the 12-month survival rate was 0.28 when death was defined as failure to reduce IOP by 20% or more below preoperative levels. Elahi et al. reported a better 1-year survival rate at 0.44 after PSLT using the same definition used in our study [10]. However, in the present study, PSLT was administrated in more advanced stages characterized by a larger number of OHMs and more progressed visual field impairment, while in the previous reports, the average mean defect (MD) was about 5 dB in HFA perimetry, which indicates a relatively early stage of glaucoma. Ahuja et al. found that 28% of patients with advanced glaucoma undergoing ab interno trabeculotomy using the trabectome required additional intervention compared to only 10% of patients with mild to moderate glaucoma [13]. In the advanced stage of glaucoma, additional open glaucoma surgeries are often required earlier when lowering IOP is not prompt or insufficient, which might have affected the survival rate in the present study. The drop-out group showed significantly higher baseline IOP and eye drop scores than the non-drop-out group, indicating that PSLT may be more suitable for those in less advanced stages.

For patients being treated with maximized OHM, open glaucoma surgery may be preferable and effective in lowering IOP and maintaining the visual field [1]. However, immediate open glaucoma surgery may not be possible in several situations, such as a poor systemic condition of the patient or the coronavirus pandemic, and PSLT is very important as an alternative intervention when open surgery cannot be performed immediately. Another advantage of PSLT is that there are relatively fewer complications with the procedure. In the present study, only one eye showed a transient increase in IOP exceeding 5 mmHg compared to the preoperative level, and no other adverse events, such as anterior iris adhesion, were observed in any patient. The present results showed that, although the effect of PSLT of lowering IOP by more than 20% was limited for the OAG patients receiving maximized OHMs, it could be expected to reduce IOP by more than 10% in 57.5% of the cases over 12 months. Hence, in certain cases, PSLT may be considered a stopgap until the next open surgery.

The limitations of this study are its research design, which was of a retrospective nature, and the small sample size. The results of the present study warrant a prospective study with a larger sample size. In addition, the cohort in this study included both primary OAG and secondary OAG (namely, PEG) cases, which did not distinguish the effects of PSLT on each phenotype. Moreover, a quantitative analysis in visual fields was not conducted, since half of the cases were measured for the visual field with Goldmann perimetry. Further studies are required in order to clarify those issues.

In conclusion, PSLT could be considered an adjunctive treatment for patients with advanced glaucoma stages who require many OHMs, although 22.5% of the treated eyes required subsequent surgical interventions.

## Figures and Tables

**Table 1:** Baseline characteristics of the patients.

Sex	male 19, female 14
Age (years)	72.7 ± 10.7 (range 57–89)
Type of glaucoma	POAG 35 eyes, PEG 5 eyes
Lens status	Phakic 17 eyes, IOL 23 eyes
IOP (mmHg)	20.1 ± 4.9
Eye drop score	4.1 ± 1.1

POAG: primary open angle glaucoma, PEG: pseudoexfoliation glaucoma, IOL: intraocular lens, IOP: intraocular pressure.

**Table 2:** The mean survival time (months) when the rate of decrease in IOP was less than 10% (top) or 20% (bottom) on two consecutive occasions.

		95% Confidence Interval	
Estimate	Standard Error	Lower Bound	Upper Bound
8.125	0.779	6.599	9.651
		95% Confidence Interval	
Estimate	Standard Error	Lower Bound	Upper Bound
4.925	0.773	3.410	6.440

## Data Availability

All data generated or analyzed during this study are included in this article.
